# The role of Phafin proteins in cell signaling pathways and diseases

**DOI:** 10.1515/biol-2022-0896

**Published:** 2024-06-27

**Authors:** Tuoxian Tang, Jing Sun, Chen Li

**Affiliations:** Department of Biology, University of Pennsylvania, Philadelphia, Pennsylvania, United States of America; Department of Biostatistics and Epidemiology, Drexel University, Philadelphia, Pennsylvania, United States of America; Department of Biology, Chemistry, Pharmacy, Free University of Berlin, Berlin, Germany

**Keywords:** Phafin1, Phafin2, Pib2, idiopathic pulmonary fibrosis, cancer

## Abstract

Membrane-associated proteins are important membrane readers that mediate and facilitate the signaling and trafficking pathways in eukaryotic membrane-bound compartments. The protein members in the Phafin family are membrane readers containing two phosphoinositide recognition domains: the Pleckstrin Homology domain and the FYVE (Fab1, YOTB, Vac1, and early endosome antigen 1) domain. Phafin proteins, categorized into two subfamilies, Phafin1 and Phafin2, associate with cellular membranes through interactions involving membrane-embedded phosphoinositides and phosphoinositide-binding domains. These membrane-associated Phafin proteins play pivotal roles by recruiting binding partners and forming complexes, which contribute significantly to apoptotic, autophagic, and macropinocytotic pathways. Elevated expression levels of Phafin1 and Phafin2 are observed in various cancers. A recent study highlights a significant increase in Phafin1 protein levels in the lungs of idiopathic pulmonary fibrosis patients compared to normal subjects, suggesting a crucial role for Phafin1 in the pathogenesis of pulmonary fibrosis. Additionally, phosphatidylinositol-3-phosphate-binding 2 (Pib2), a close relative of the Phafin1 protein, functions as an amino acid sensor activating the TOCR1 pathway in yeasts. This review focuses on delineating the involvement of Phafin proteins in cellular signaling and their implications in diseases and briefly discusses the latest research findings concerning Pib2.

## Graphical abstract

**Figure:**
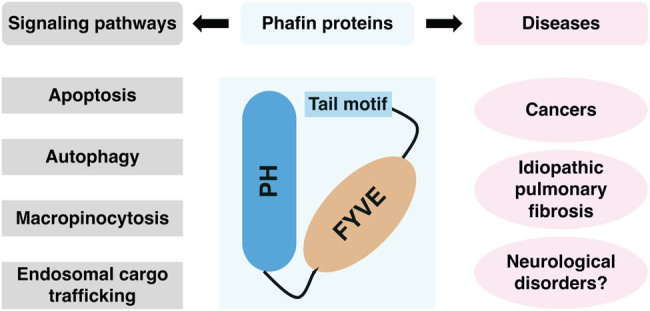

## 1 Introduction

Membrane proteins are broadly classified into integral and peripheral proteins. Constituting approximately 5% of all human proteins, peripheral membrane proteins are reversibly associated with the lipid bilayer of cellular membranes in a spatiotemporally dependent manner [1,2]. Typically, these peripheral membrane proteins contain lipid-binding domains that recognize specific lipids in the membrane which drive the transient association to a specific membrane. The membrane-associated proteins serve as adaptors and regulatory proteins, rendering them vital in cell signaling and trafficking pathways [3]. About 30 peripheral membrane proteins are currently targeted in drug discovery as the lipid–protein interaction can be potentially regulated [3,4].

In 2005, Phafins were initially defined as proteins containing both Pleckstrin Homology (PH) and Fab1, YOTB, Vac1, and EEA1 (FYVE) domains. The Phafin protein family consists of two subfamilies: Phafin1 and Phafin2 [5]. Phafin1 is also known as LAPF (a lysosome-associated apoptosis-inducing protein containing PH and FYVE domains) or PLEKHF1 (pleckstrin homology and FYVE domain containing 1). Phafin2 is also referred to as EAPF (an endoplasmic reticulum (ER)-associated apoptosis-involved protein containing PH and FYVE domains) and PLEKHF2 (pleckstrin homology and FYVE domain containing 2) [6]. Early studies have identified Phafins as pro-apoptotic proteins. Phafin1 is a lysosome-associated protein that recruits phosphorylated p53 to lysosomes and induces lysosomal membrane permeabilization in apoptosis [7]. Phafin2 is an ER-associated protein that facilitates tumor necrosis factor-alpha (TNF-α)-induced cellular apoptosis through the ER–mitochondrial apoptotic pathway [8].

Recent years have brought forth numerous exciting discoveries regarding the Phafin protein family. After induction of autophagy, Phafin2 was associated with the lysosomal membrane through PtdIns(3)P-binding, and the Phafin2–Akt complex accumulated in the lysosome, which indicated that Phafin2 is involved in autophagy [9]. Both the Phafin2 PH domain and the Phafin2 FYVE domain bind PtdIns(3)P but exhibit distinct PtdIns(3)P-binding properties. An autoinhibition mechanism was identified in Phafin2, where the Phafin2 FYVE domain constitutively binds to PtdIns(3)P, while the Phafin2 PH domain’s PtdIns(3)P-binding is autoinhibited by the conserved C-terminal polyD motif of Phafin2 [10]. A recent study reported Phafin2 as a coincidence detector of PtdIns(3)P and PtdIns(4)P, promoting the internalization and maturation of macropinosomes by coordinating the actin dynamics [11]. Phafin1 has been implicated in the activation of M2 macrophage and exacerbation of pulmonary fibrosis [12]. Moreover, significant progress has been made in the research on a Phafin1-like protein, Pib2, which is identified as a critical regulator of yeast target of rapamycin complex 1 (TORC1) [13]. In this review, we provide a summary of major findings and unanswered questions about the Phafin protein family, with a focus on their roles in signaling pathways and implications in diseases. The latest findings on Pib2 are also briefly discussed due to its close resemblance to Phafin1.

## 2 Structural features of Phafin proteins and Pib2

### 2.1 Phafin proteins have two phosphoinositide-binding domains

**Figure 1:**
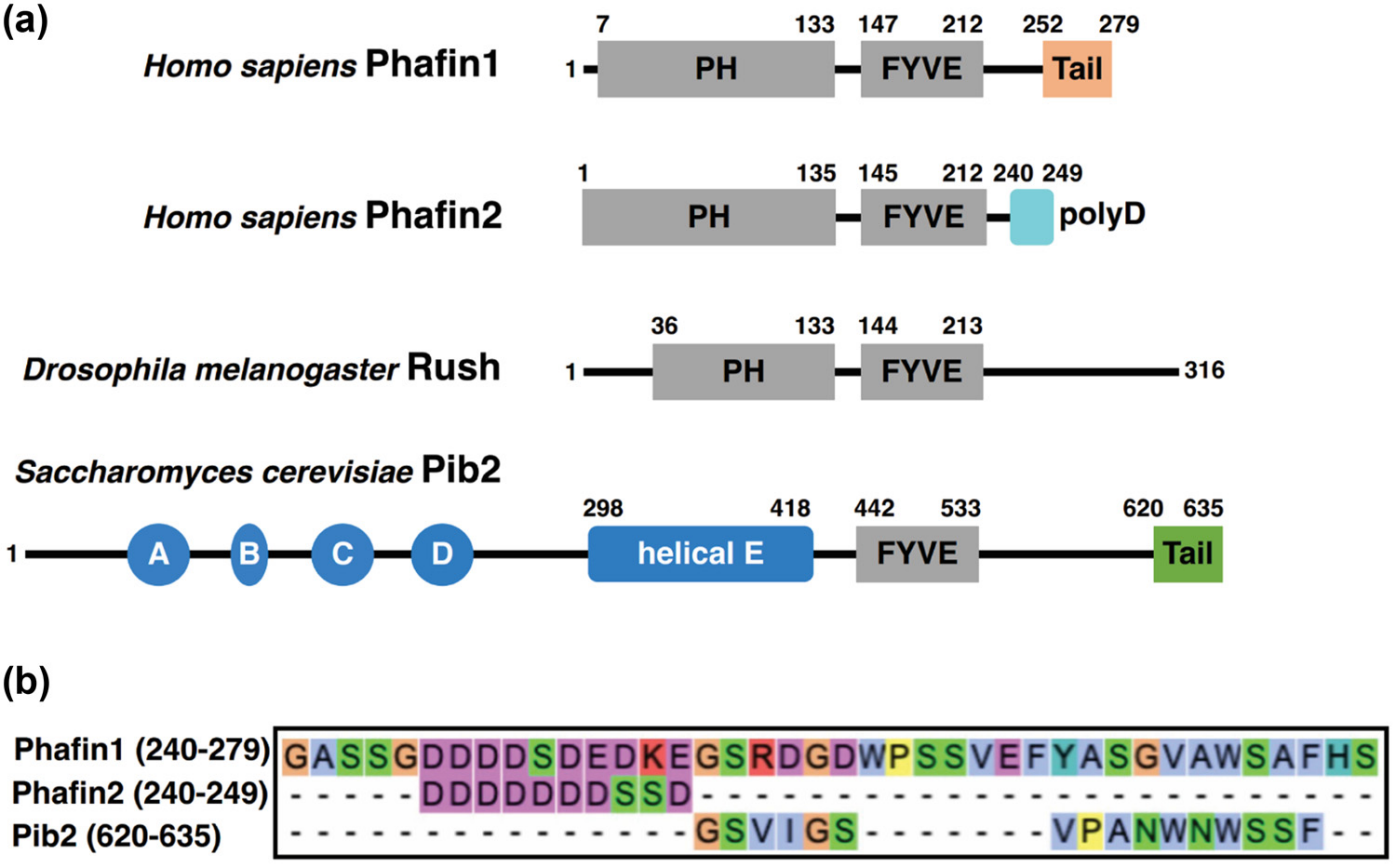
Modular organization of Phafin proteins and Pib2. (a) Schematic diagram of full-length Phafin1, Phafin2, Rush, and Pib2 with defined domains and motifs. (b) Sequence alignment analysis of C-terminal regions of Phafin proteins and Pib2. The C-terminal region of human Phafin1 (240–279 aa), the polyD tail of human Phafin2 (240–249 aa), and the C-terminal domain of Pib2 (620–635 aa) were aligned using the Clustal Omega Multiple Sequence Alignment program.

Phafin proteins are distinguished by the presence of two phosphoinositide-binding domains in their structural elements: the N-terminal PH domain, comprising approximately 120 amino acid residues, and the central FYVE domain, consisting of about 70 amino acid residues (Figure 1a). Rush, a Drosophila homolog of human Phafin1 and Phafin2, exhibits a similar modular organization [14]. The Rush protein is longer than human Phafin1 and Phafin2, containing 316 amino acid residues. Pib2, a relative of Phafin1, is an FYVE domain-containing protein that lacks the PH domain. The other conserved regions of Pib2 include the N-terminal A–D motifs, a helical-E region, and a C-terminal tail (Figure 1a and b) [15,16].

**Figure 2:**
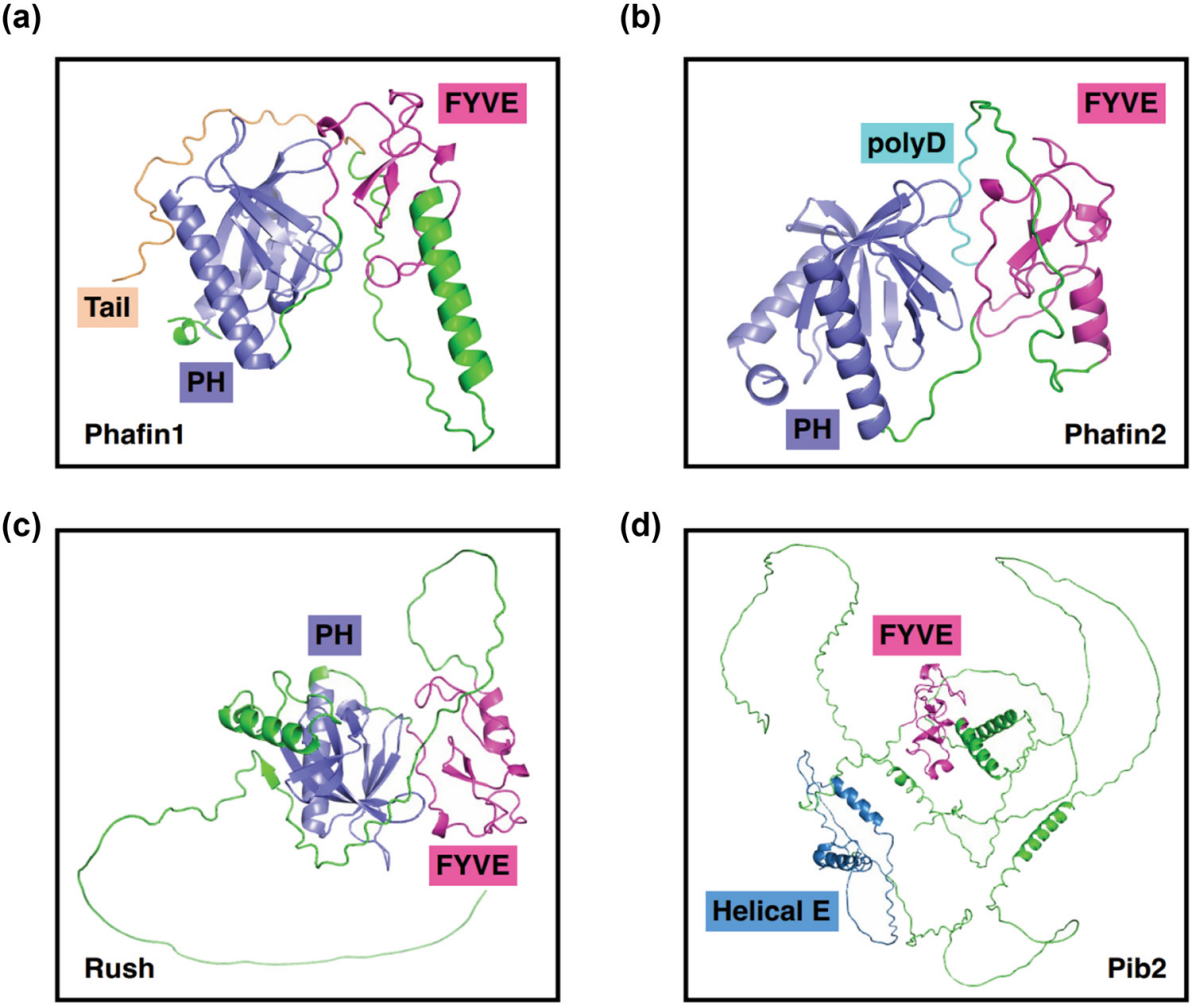
Predicted protein structures of Phafin proteins and Pib2. Predicted protein structures of Phafin1 (a), Phafin2 (b), Rush (c), and Pib2 (d). The protein structures were predicted using AlphaFold2. Following protein sequences were used to build the protein structures: *Homo sapiens* Phafin1, UniProt accession number Q96S99; *Homo sapiens* Phafin2, UniProt accession number Q9H8W4; *Drosophila melanogaster* Rush, UniProt accession number O76902; *Saccharomyces cerevisiae* Pib2, UniProt accession number P53191.

The structures of Phafin proteins and Pib2 have not yet been determined. Our structural analysis of Phafin2 has provided valuable insights into the structural features of Phafin proteins. The human Phafin2 is a moderately elongated monomer with an estimated molecular weight of approximately 28 kDa. Circular dichroism analysis suggests that Phafin2 adopts an α/β structure, with an estimated secondary structural content of 23% α-helix, 21% β-strand, 15% β-turn, and 41% random coil [17,18]. Herein, the protein structures of Phafin1, Phafin2, Rush, and Pib2 were predicted using AlphaFold2, an artificial intelligence program used for protein structure prediction (Figure 2a–d) [19]. The N-terminal PH domain exhibits a seven-stranded β-sheet sandwich with a long α-helix and the central FYVE domain displays a pair of two-stranded β-sheets and a small α-helix. Notably, in comparison with the structures of Phafin1, Phafin2, and Rush, Pib2 demonstrates a more relaxed structural arrangement, with a significant portion represented by the random coil (Figure 2a–d). However, it is necessary to point out that predicted protein structures may not accurately represent the real structures of Phafin proteins and Pib2, warranting further structural studies to decipher their structural features and protein–ligand interactions.

The association between Phafin proteins and cellular membranes was mediated by their phosphoinositide-binding domains. Both Phafin1 and Phafin2 showed a preference for PtdIns(3)P binding in the lipid–protein overlay assay [20,21]. PtdIns(3)P, or phosphatidylinositol 3-phosphate, is one of the seven phosphoinositides, a group of phospholipids characterized by phosphorylation at the 3-, 4-, and/or 5-positions of the inositol ring. Phosphoinositides are differentially distributed in cellular membranes, playing key roles in cell signaling transduction and membrane remodeling [22,23]. PH domains and FYVE domains exhibit distinct phosphoinositide-binding specificities. The Phafin2 PH domain was shown to bind PtdIns(3)P, PtdIns(4)P, and PtdIns(5)P, while the Phafin2 FYVE domain specifically binds PtdIns(3)P [9,11].

### 2.2 The C-terminal tail region is a functional motif

In addition to the two conserved phosphoinositide-binding domains, the C-terminal regions of Phafin proteins serve as functional motifs. Specifically, the C-terminal tail of Phafin1 functions as a lysosomal targeting signal. In studies, the wild-type Phafin2, lacking this C-terminal tail, and the Phafin1 mutant with the tail domain deleted, were unable to associate with lysosomes [20]. Similar to Phafin1, Pib2 also possesses a functional C-terminal motif, and there is a notable similarity between their amino acid sequences, which explains why Pib2 is considered a close relative of Phafin1 rather than Phafin2, despite all three sharing a conserved FYVE domain (Figure 1a and b) [15]. It has been reported that the Pib2 tail motif plays a role in TORC1 activation [24,25]. Further details regarding the role of Pib2 in TORC1 regulation will be provided in Section 3.3.

**Figure 3:**
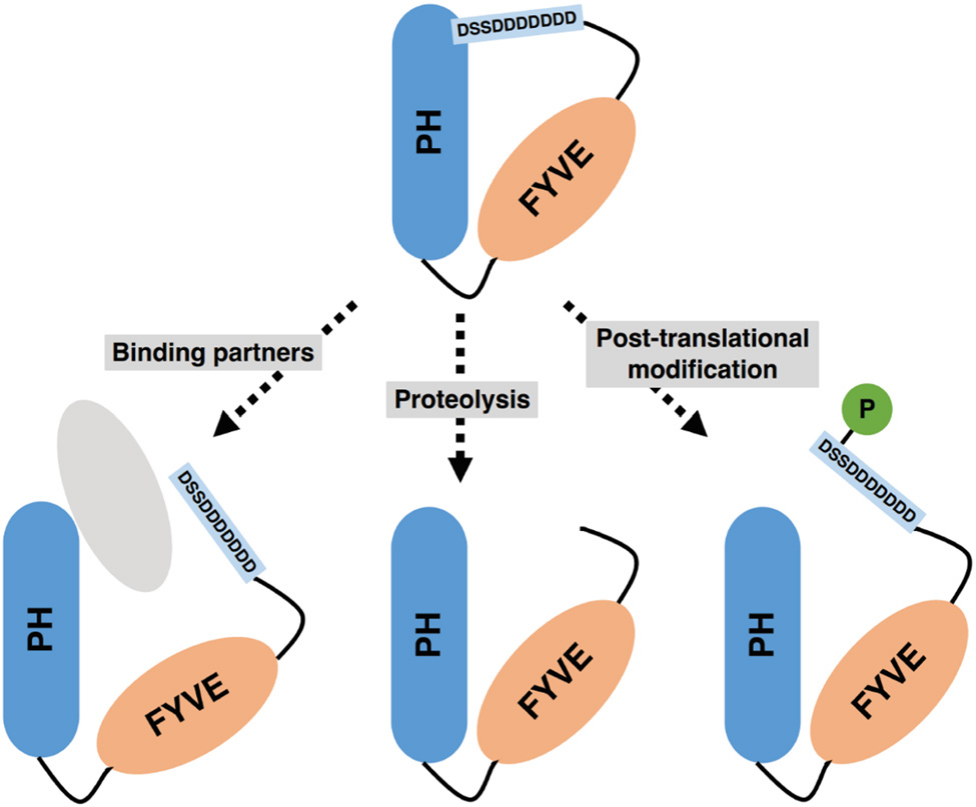
Proposed autoinhibition-relief mechanisms of Phafin2.

Phafin2 is 30 amino acid residues shorter than Phafin1, as depicted in Figure 1a. Consequently, the amino acid residues beyond the central FYVE domain constitute a shorter C-terminal region. Within the C-terminus of Phafin2 (Figure 1b), a motif rich in aspartic acid residues, referred to as polyD (corresponding to residues 240–249 of Phafin2), was identified. This polyD motif is conserved among Phafin proteins across various species [10]. The polyD motif was shown to intramolecularly inhibit the Phafin2 PH domain’s PtdIns(3)P-binding without disrupting the Phafin2 FYVE domain’s PtdIns(3)P-binding, which represents an autoinhibition mechanism in Phafin2 (Figure 3) [10]. Our unpublished data suggest that the polyD peptide directly interacts with the Phafin2 PH domain, causing perturbations of some PH domain resonances in the NMR spectra. In contrast, the Phafin2 FYVE domain does not interact with the polyD motif, indicating that this motif specifically targets the Phafin2 PH domain [26].

Autoinhibition is a molecular mechanism by which proteins employ inhibitory modules to regulate the function of other domains through intramolecular interactions [27]. This widespread regulatory strategy plays a significant role in modulating the DNA binding of transcription factors, regulating enzyme activity, and controlling the cellular localization of proteins. Together with counteracting measures to relieve autoinhibition, it adds an extra layer of regulation to cellular systems [27,28]. Functionally, the Phafin2 polyD motif regulates the lipid-binding activity of the PH domain, suppressing excessive membrane association [11]. Autoinhibition in Phafin2 can potentially be alleviated through various mechanisms, including binding to other partners, post-translational modifications, and partial proteolysis of the polyD motif (Figure 3).

Previous studies have identified several binding partners of Phafin2, including Akt (protein kinase B) [9], early endosome antigen 1 (EEA1) [29], and JNK-interacting protein 4 (JIP4) [30]. Interaction with these proteins may induce conformational changes, leading to the relief of autoinhibition. Three residues S239, S247, and S248 within the polyD region of Phafin2 are available for phosphorylation [31], which may present an additional mechanism to counteract the autoinhibition. Further studies are necessary to elucidate how the autoinhibition mechanism in Phafin2 is regulated. It is worth noting that Phafin1 also exhibits an aspartic acid residue-rich motif, followed by additional amino acid residues. However, it remains unknown whether this motif plays regulatory roles in Phafin1 proteins.

## 3 Phafin proteins and Pib2 in cell signaling pathways

### 3.1 Role of Phafin1 in apoptosis and autophagy

Apoptosis, a process of programmed cell death, is employed by cells to eliminate unwanted cells during development and aging [32,33]. Phafin1 is a proapoptotic protein, acting as an adaptor that recruits phosphorylated p53 to the lysosomal membrane. The formation of phosphorylated p53 and Phafin1 complex on the lysosomal membrane triggers lysosomal membrane permeabilization and activates the apoptotic pathway. Phafin1 specifically interacts with phosphorylated p53 through its PH domain. Silencing Phafin1 expression hinders the lysosomal translocation of phosphorylated p53, resulting in reduced lysosomal membrane permeabilization and apoptosis [7].

Increased expression of Phafin1 in HEK 293T cells led to the formation of enlarged vesicles and the regulation of certain receptors’ density on the cell membrane, indicating that Phafin1 is involved in endocytosis. Similarly, overexpression of the endosome-associated Rush in *Drosophila* follicular cells resulted in the enlargement of late endosomes and disrupted the progression of endosomal cargoes [14].

Phafin1-containing vesicles in the cells exhibited positive signals for LysoTracker, a fluorescent dye used for labeling lysosomes, suggesting that Phafin1 is targeted to the lysosomes. Co-transfection of Phafin1 and hLC3A into cells was able to induce autophagy. Autophagy, or macroautophagy, represents a highly conserved eukaryotic cellular digestion mechanism in which cells break down unnecessary or dysfunctional components through lysosomes, subsequently recycling the resulting molecules [34,35]. The C-terminal tail of Phafin1 played a crucial role in lysosomal association and autophagy induction, as a tail domain deletion mutant of Phafin1 failed to associate with lysosomes or induce autophagy [20]. Phafin1 has been identified as an autophagy-related biomarker in dilated cardiomyopathy using machine learning algorithms and bioinformatics tools [36].

### 3.2 Role of Phafin2 in cell signaling pathways

#### 3.2.1 Apoptosis

**Figure 4:**
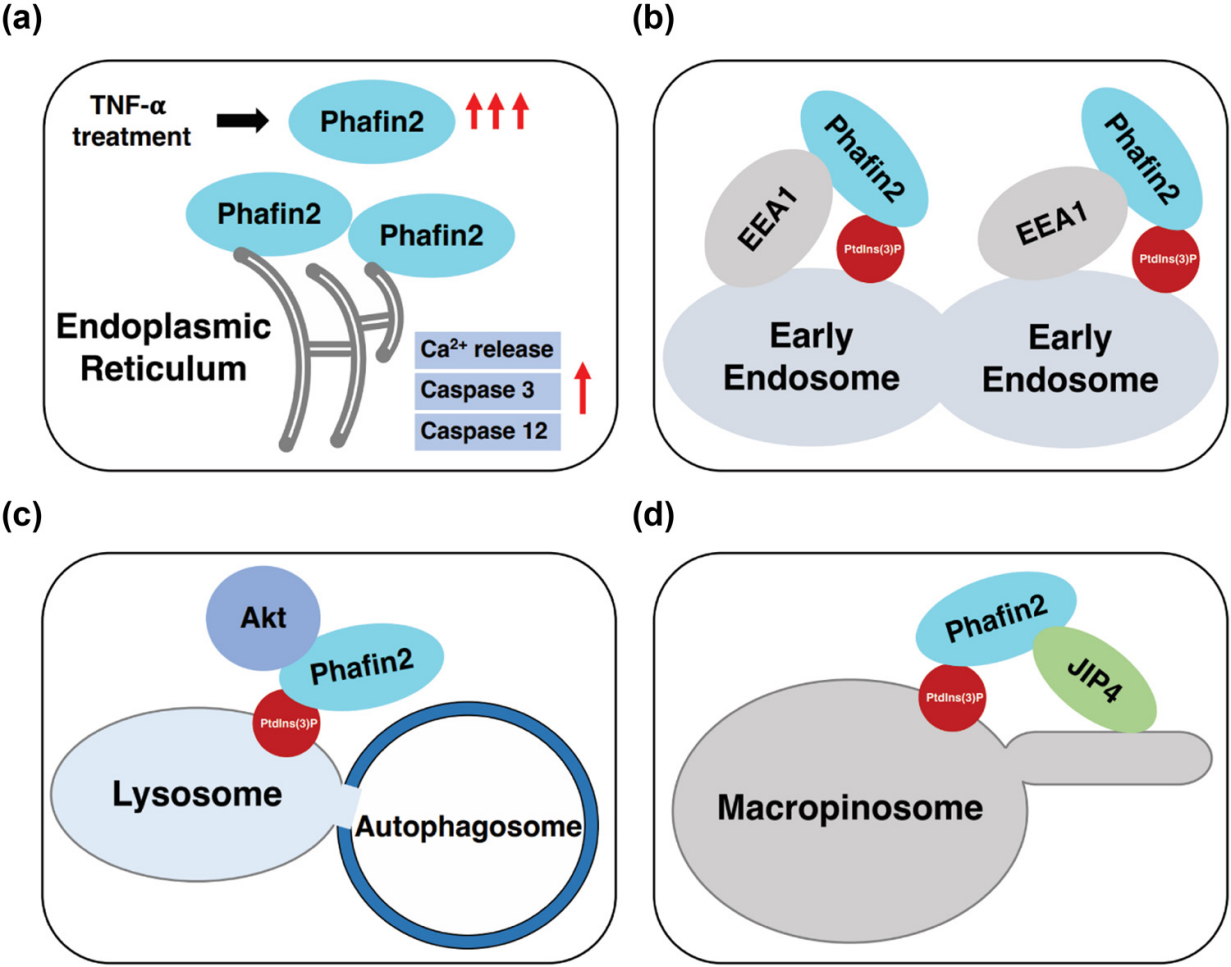
Role of Phafin2 in cell signaling pathways. (a) Phafin2 is a pro-apoptotic protein. (b) Phafin2 promotes endosomal fusion. (c) Formation of the Phafin2–Akt complex on lysosomes is a critical step in autophagy. (d) Phafin2 facilitates macropinocytotic membrane tubulation in orchestration with JIP4.

Like Phafin1, Phafin2 also functions as a pro-apoptotic protein. A proposed model elucidates the role of Phafin2 in enhancing TNF-α-induced cellular apoptosis: Phafin2 expression levels increase upon TNF-α treatment; Phafin2 partially translocates to the ER through its phospholipid-binding and becomes locally activated; this leads to enhanced calcium efflux from the ER, disrupting Ca^2+^ homeostasis; the perturbed Ca^2+^ balance activates ER-resident caspase 12 and caspase 3 cascades, promoting TNF-α-triggered mitochondrial membrane permeabilization and the release of apoptosis-inducing factors (Figure 4a) [8].

#### 3.2.2 Endosomal cargo trafficking

Both Phafin1 and Phafin2 are endosome-associated proteins, playing crucial roles as regulators in the endosomal trafficking pathway. The membrane association is primarily mediated by interactions between the FYVE domain of Phafin proteins and PtdIns(3)P, a phosphoinositide abundant in endosomes. Overexpression of Phafin2 leads to the formation of enlarged endosomes, while depletion of Phafin2 decreases the size of early endosomes [21,29]. In cells transfected with Phafin2, the elevated expression precludes the internalization of insulin receptors, resulting in a higher density of insulin receptors on the membrane compared to control cells [21]. Phafin2 strongly colocalizes with EEA1 on the endosome membrane and is identified as an interactor of EEA1. It regulates the trafficking of epidermal growth factor receptors by modulating endosome fusion in coordination with EEA1 (Figure 4b) [29].

#### 3.2.3 Autophagy

Phafin proteins lack transmembrane domains, indicating they are not membrane proteins. Under normal conditions, Phafin2 is diffusely spread in the cytosol. Upon autophagy induction by rapamycin or Hank's balanced salt solution treatment, Phafin2 co-localizes with Akt on lysosomes. The lysosomal accumulation of the Akt–Phafin2 protein complex is enhanced after autophagy induction. Phafin2’s association with lysosomes depends on its interactions with PtdIns(3)P. A PtdIns(3)P interaction-defective mutant of Phafin2 failed to associate with the lysosomal membrane, and lysosomal co-localization of the Phafin2–Akt complex was disrupted. Both Phafin2 and Akt play crucial roles in autophagy induction. Mouse macrophages transfected with Phafin2-siRNA or Akt-siRNA failed to initiate the autophagic process (Figure 4c) [9].

#### 3.2.4 Macropinocytosis

Macropinocytosis is an actin-dependent cellular mechanism utilized by cells to take up extracellular fluids and soluble macromolecules, leading to the formation of large vesicles known as macropinosomes [37,38]. Newly formed macropinosomes are surrounded by a dense actin network. Phafin2 was shown to escort the nascent macropinosomes through a coincidence detection of PtdIns(3)P and PtdIns(4)P. Direct binding of Phafin2 to actin through its PH domain facilitates the shedding of the actin matrix, promoting the maturation of macropinosomes [11]. In addition, Phafin2 recruits JIP4 to tubulating macropinosomes in a PtdIns(3)P-dependent manner. The direct interaction between Phafin2 and JIP4, mediated by the Phafin2 PH domain, targets JIP4 to macropinosome tubules, facilitating the formation of membrane tubules (Figure 4d) [30].

### 3.3 Pib2 is a TORC1 regulator

TORC1, a universally conserved protein complex in eukaryotes, serves as a crucial regulator of cell growth in response to nutrients, particularly amino acids. Under nutrient-rich conditions, activated TORC1 promotes anabolic reactions such as protein synthesis while inhibiting catabolic processes like autophagy in cells [39]. A well-established regulator of TORC1 in yeasts is the heterodimeric small GTPase complex Gtr1–Gtr2 (mammalian orthologs Rag A/B and Rag C/D, respectively). The Gtr1–Gtr2 complex associates with the vacuolar membrane through the Ego scaffolding protein complex, consisting of Ego1, Ego2, and Ego3 [40–43].

Pib2 has been identified as a significant TORC1 regulator in the budding yeast *Saccharomyces cerevisiae* [15,24]. The Pib2 FYVE domain plays a crucial role in its association with the vacuole membrane. A truncated Pib2 mutant lacking the FYVE domain failed to bind PtdIns(3)P, preventing proper association with the vacuole membrane. Different regions of Pib2 exhibit antagonistic effects on the TORC1 regulation: the N-terminus inhibits TORC1, while the C-terminus activates TORC1 [16,24]. Pib2 has been reported to play a role in glutamine-responsive TORC1 activation, regulating TORC1 signaling independently of the Ego complex. Acting as a glutamine sensor, Pib2 directly activates TORC1 [25,40,42].

**Figure 5:**
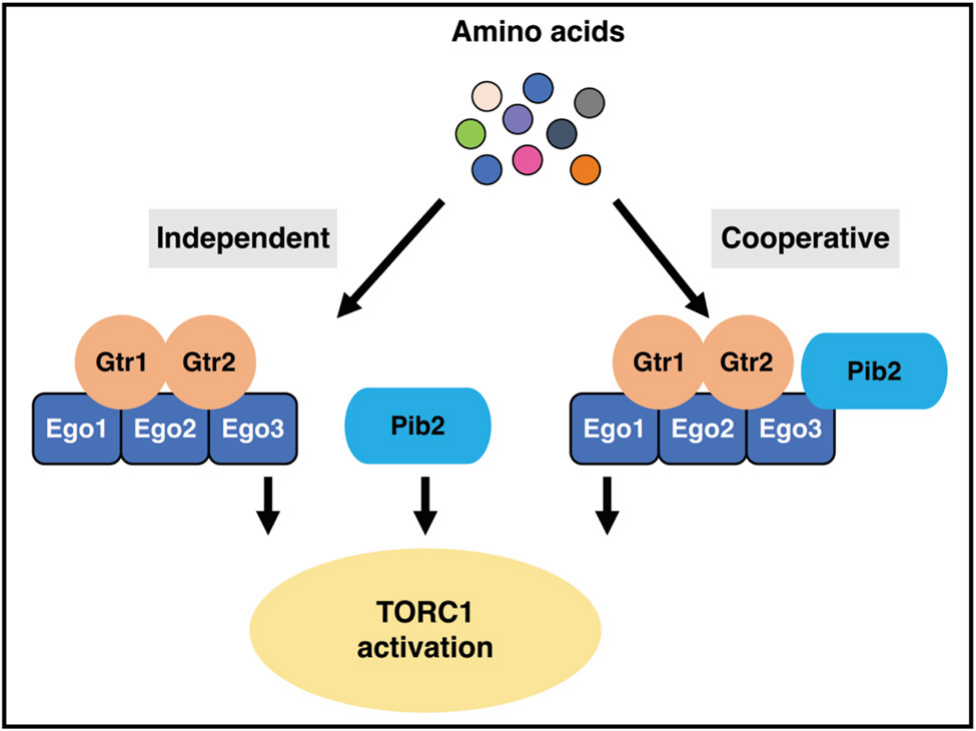
Two models for TORC1 activation by Pib2 and Gtr 1/2 in *S. cerevisiae*.

However, conflicting findings arise from another study proposing that both Pib2 and the Ego complex are required for TORC1 activation [43]. Pib2 and the Ego complex synergistically function in the same pathway, resulting in the activation of TORC1 signaling. The discrepancy in understanding the relationship between Pib2 and the Ego complex may be attributed to different experimental settings, which include variations in yeast strains, TORC1 activation stimuli, and TORC1 activity assays. A recent study may offer a possible explanation for this discrepancy, suggesting that Pib2 functions as a cysteine sensor in TORC1 regulation and different amino acids exhibit distinct dependencies on the Gtr and Pib2 pathways [44]. The relationship between the Gtr pathway and the Pib2 pathway in TORC1 regulation is currently contentious and requires further investigation (Figure 5). A dual-phase TORC1 activation model was proposed to reconcile these distinct observations. In Phase-1 (characterized by an acute and transient pulse), Pib2 and the EGO complex synergistically function together, while in Phase-2 (marked by a slow and continuous activation), they work independently [13].

## 4 Phafin proteins in diseases

### 4.1 Cancers

The expression of Phafin proteins is elevated in various cancers, including hepatocellular carcinoma [21], breast cancer [45–47], ovarian cancer [48], osteosarcoma [49], hepatoid adenocarcinoma of the stomach [50], and hereditary non-polyposis colorectal cancer [51] (Table 1). Analysis of a public hepatocellular carcinoma dataset revealed a significantly higher Phafin2 mRNA level in human hepatocellular carcinoma tissues compared to normal livers [21]. Both Phafin1 and Phafin2 are upregulated in breast cancer [45,47]. Phafin1 was identified as a macrophage-related hub gene that is important for the prognosis of triple-negative breast cancer, although its functional annotations are not available [46].

**Table 1:** Phafin proteins in diseases

Phafin proteins	Diseases	Short description	References
Phafin2	Hepatocellular carcinoma	Phafin2 mRNA level is significantly higher than normal cells	[21]
Phafin2	Breast cancer	Expression profile specifically marks estrogen receptor (ERα)-positive breast cancer cells	[47]
Phafin1	Breast cancer	Amplified and overexpressed in ERα-negative grade III breast cancer	[45]
Phafin1	Breast cancer	Identified as a hub gene that is associated with macrophages in triple-negative breast cancer	[46]
Phafin1	Ovarian cancer	Identified as one of the genes in a prognostic 11-gene signature for ovarian cancer	[48]
Phafin1	Osteosarcoma	Identified as one of the six genes in a prognostic risk score model for osteosarcoma patients	[49]
Phafin1	Hepatoid adenocarcinoma of the stomach (HAS)	A frequently amplified gene in HAS tumor tissues	[50]
Phafin1	Hereditary non-polyposis colorectal cancer (HNPCC)	Increased expression in HNPCC samples vs controls	[51]
Phafin1	IPF	Upregulation in IPF patients; regulate macrophage M2 activation	[12]
Phafin1	Dilated cardiomyopathy	A diagnostic autophagy-related biomarker of dilated cardiomyopathy	[36]
Phafin1	Chronic graft versus host disease (cGVHD)	An RNA biomarker that can distinguish cGVHD cases from non-cGVHD controls	[55]
Phafin1	Diabetes mellitus-related atherogenesis	Upregulation in diabetes mellitus-associated coronary heart diseases	[56]

It is crucial to note that all studies demonstrating the upregulation of Phafin proteins or amplification of *phafin* genes in cancers rely on bioinformatics tools for the identification of disease-related genes. The functions of Phafin proteins in cancers have remained elusive. The elevated expression levels observed in cancers may suggest that Phafin proteins confer an advantage for the growth of cancer cells. Phafin proteins play a role in autophagy, apoptosis, and macropinocytosis – cellular pathways that cancer cells exploit to promote cellular proliferation, withstand microenvironmental stress, enhance resistance to anti-tumor drugs, and facilitate metastasis [52–54]. It has been hypothesized that the overexpression of Phafin2 could be advantageous to cancer cells, possibly owing to its involvement in the macropinocytotic pathway, which supports nutrient scavenging by cancer cells [11].

### 4.2 Idiopathic pulmonary fibrosis (IPF)

In addition to its upregulation in cancers, Phafin1 has been identified as an overexpressed protein or an amplified gene in various diseases, including IPF [12], dilated cardiomyopathy [36], chronic graft versus host disease [55], and diabetes mellitus-related atherogenesis [56]. A recently published article has shed light on the role of Phafin1 in IPF, the most prevalent type of interstitial lung disease characterized by lung scarring, leading to symptoms such as dry cough, extreme fatigue, and shortness of breath [12,57]. Phafin1 appears to play a role in the pathogenesis of IPF and holds potential as a therapeutic target for its treatment. The protein level of Phafin1 is approximately 2.5 times higher in IPF patients compared to control subjects. Moreover, Phafin1 is highly expressed in macrophages in mice with bleomycin-induced pulmonary fibrosis. The increased expression of Phafin1 promotes M2 macrophage activation through enhanced PI3K/Akt signaling. Utilizing *phafin1* siRNA-loaded liposomes to suppress Phafin1 protein expression resulted in a significantly reduced number of M2 macrophages and mitigated lung fibrosis in mice. This approach may represent a potential treatment strategy for IPF [12].

### 4.3 In response to environmental factors and treatments

Phafin proteins act as adaptor proteins in apoptotic, autophagic, endosomal trafficking, and macropinocytotic signaling pathways, yet their functions may extend beyond current exploration. Phafin2, for instance, has been recognized as an antibacterial effector in zebrafish embryos, playing a pivotal role in the nonspecific immune defense of fish [58].

The *phafin1* gene exhibits upregulation in the striatum, hypothalamus, or hippocampus when mice or rats are exposed to external environmental factors or various treatments, such as early-life stress [59,60], drugs of abuse [61,62], glucocorticoids [63,64], and Levosimendan treatment [65] (Table 2). However, the reasons behind the altered expression levels of the *phafin1* gene in response to these environmental factors and treatments remain unclear. Notably, the *phafin2* gene expression did not show differential regulation in these studies, suggesting that Phafin1 and Phafin2 may serve distinct functions in animals. Given the upregulation of the *phafin1* gene in brains, it becomes intriguing to explore the function of Phafin1 in the central nervous system.

**Table 2:** Upregulation of the *phafin1* gene in response to environmental factors/treatments

Environmental factors/treatments	Short description	References
Early life stress	Upregulation in mouse prefrontal cortex	[59,60]
Drugs of abuse	Upregulation in mouse striatum	[61]
Opioid dependence	Upregulation in mouse striatum	[62]
Glucocorticoid	Upregulation in mouse hippocampus	[63]
Glucocorticoid	Upregulation in rat hypothalamus	[64]
Levosimendan treatment	Upregulation in Levosimendan-treated diabetic rats after myocardial infarction	[65]
Exploratory locomotion	*phafin1* as a candidate gene for exploratory locomotion traits	[68]
Eyeblink conditioning	Upregulation in the early-stage learning	[69]
Chronic unpredictable mild stress	Upregulation in mouse hippocampus	[70]

## 5 Conclusions and future directions

This review article outlines the domain structure of Phafin proteins, provides a concise summary of their involvement in cellular signaling pathways, and delves into their diverse functions in diseases. Phafin proteins are characterized by two phosphoinositide-binding domains, namely PH and FYVE. Besides, the C-terminal tail emerges as a functional domain. Significant progress has been made in this protein family, but there is still ample opportunity for further research to uncover new findings.

Pib2 (a relative of human Phain1), known for regulating TORC1, assumes a critical role in nutrient sensing in yeasts. Structurally, both Phafin1 and Pib2 contain an FYVE domain and a C-terminal tail. Furthermore, they share some functional similarities, converging on the TORC1 as Phafin1 is involved in apoptosis and autophagy, cellular processes modulated by TORC1. An intriguing question remains regarding whether Phafin1 participates in TORC1 regulation in mammalian cells. To address this issue, it is imperative to develop Phafin1 knock-down or knock-out cell models.

An autoinhibition mechanism has been discovered in Phafin2, wherein the C-terminal polyD motif intramolecularly interacts with its PH domain, thereby regulating phosphoinositide binding. The potential unraveling of Phafin2’s protein structure could shed light on the intricacies of how the polyD tail specifically interacts with the PH domain, providing insights into the regulation of this autoinhibition mechanism in cells and its coordination in signaling pathways.

Phafin proteins are implicated in apoptosis, autophagy, endosomal cargo trafficking, and macropinocytosis – cellular pathways utilized by cancer cells for growth advantage. The upregulation of Phafin protein expression in various cancers prompts the need for further studies to explore the roles of Phafin proteins in cancers. As peripheral membrane proteins, Phafin proteins transiently associate with cellular membranes via two phosphoinositide-binding domains. Given the significance of lipid–protein interactions in drug development [66,67], further studies are warranted to elucidate their lipid interactions, which could serve as potential therapeutic targets.

Moreover, the upregulation of the *phafin1* gene in the brains of mice or rats responding to various environmental factors and treatments raises intriguing possibilities. No studies have explored the functions of Phafin proteins in the central nervous system, presenting an untapped area that could yield insightful and unexpected results.
